# Excitation‐Selective Hydrochromic Upconversion in Zero‐Dimensional Metal Halides

**DOI:** 10.1002/advs.78059

**Published:** 2026-09-28

**Authors:** Joo Hyeong Han, Jeong Min Seo, Sung Woo Jang, Yong Min Park, San Ha Choi, Ji Hyeon Cha, Won Bin Im

**Affiliations:** ^1^ Division of Materials Science and Engineering Hanyang University Seoul Republic of Korea

**Keywords:** hydrochromism, information encryption, moisture dosimeter, photon upconversion, stimuli‐responsive materials, zero‐dimensional metal halides

## Abstract

Smart luminescent materials that dynamically respond to external stimuli are of considerable interest for optical readout and sensing applications. Here, we show that, in Cs_3_GdCl_6_‐based zero‐dimensional metal halides, the occurrence of hydrochromic switching is determined by the excitation condition itself. In particular, Cs_3_Gd_0.8_Er_0.2_Cl_6_ shows excitation‐selective hydrochromic color evolution under moisture exposure: it retains its yellow emission under 980 nm excitation, whereas it undergoes a rapid green‐to‐red shift under 1550 nm excitation under the same conditions. Spectroscopic and kinetic analyses reveal that this selective behavior originates from excitation‐pathway‐dependent population of moisture‐sensitive excited states, with the absorption at the excitation wavelength serving as a decisive factor. This excitation‐selective behavior was further exploited to implement excitation‐selective optical readout and a power‐free sticker‐type moisture dosimeter for quantitative analysis of accumulated moisture exposure based on Fick's second law. These findings establish an excitation‐selective switching criterion for hydrochromic upconversion in zero‐dimensional metal halides and provide a design principle for excitation‐selective optical moisture readout.

## Graphical abstract

Excitation wavelength dictates whether moisture speaks. In zero‐dimensional metal halides, the same humidity leaves the upconversion emission unchanged under 980 nm excitation yet triggers a distinct green‐to‐red shift under 1550 nm. This excitation‐selective hydrochromism, governed by the absorption at each wavelength, enables stimulus‐dependent optical readout and power‐free, quantitative moisture dosimetry.

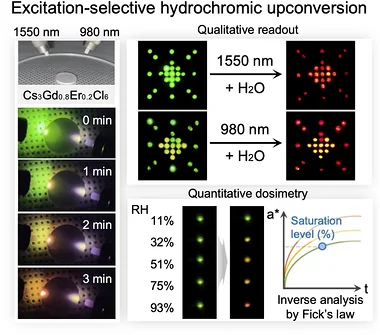

## 1 Introduction

Smart luminescent materials play a pivotal role in enabling modern optoelectronic and security systems to actively communicate or conceal information by interacting with their environment [1, 2, 3, 4, 5]. These materials can dynamically alter their physicochemical properties in response to external stimuli, including light, temperature, pressure, and moisture; therefore they can be used in high‐value applications such as anti‐counterfeiting, information encryption, and data storage [6, 7]. Recently, beyond the limitations of conventional materials that respond to only a single stimulus, there has been a growing demand for luminescent materials capable of exhibiting distinct optical responses depending on external stimuli, thereby expanding their utility in sensing and information‐related applications [8, 9].

Over the past few years, lead‐based halide perovskites have garnered significant attention as innovative materials in various optoelectronic devices such as solar cells, LEDs, and photodetectors, owing to their high photoluminescence quantum yield, color purity, and facile bandgap tunability [10, 11, 12]. Additionally, their high sensitivity to moisture, which induces structural transformations, suggests their potential application as hydrochromic sensors [13, 14, 15]. However, lead is highly toxic for humans and the environment, posing severe challenges in manufacturing and disposal; this toxicity is a major barrier to commercialization owing to stringent international regulations designed to protect the environment and human health, such as the Restriction of Hazardous Substances Directive (RoHS) [16, 17, 18]. Furthermore, the moisture instability of perovskites, which is advantageous for sensing, is a critical drawback that compromises the long‐term stability of these devices [19, 20, 21]. Consequently, the development of eco‐friendly, lead‐free metal halides that retain superior optical performances while addressing toxicity and stability issues has attracted considerable interest in materials science [22, 23]. Among these, eco‐friendly luminescent zero‐dimensional (0D) lead‐free metal halides are particularly highlighted because of their excellent chemical and thermal stabilities resulting from their unique isolated metal‐ion structural characteristics [24, 25]. In addition, low‐phonon‐energy heavy halide hosts have recently been recognized as promising platforms for lanthanide‐activated upconversion because they can suppress multiphonon relaxation and thereby enable efficient population of high‐lying excited states, expanded color tunability, and nonlinear photoactivation dynamics [26].

In this study, we investigated a Cs_3_GdCl_6_‐based zero‐dimensional lead‐free metal halide by introducing lanthanide ions (Tb^3+^, Yb^3+^, or Er^3+^). The combination of chemically stable 0D metal halide lattices with upconversion‐active lanthanide dopants provides a useful basis for designing optical materials with distinct stimulus‐dependent responses. In particular, we show that hydrochromic switching in the Er‐substituted system is not determined by moisture exposure alone, but by whether the excitation condition can sufficiently populate moisture‐sensitive excited states. By comparing 980 nm and 1550 nm excitation, we identify an excitation‐selective switching criterion for hydrochromic upconversion and clarify its mechanistic origin in terms of absorption‐dependent population of moisture‐sensitive excited states. This mechanism also provides a basis for exploiting the excitation‐selective contrast of the Er‐substituted system for both optical readout and quantitative moisture dosimetry. Thus, this study establishes excitation‐selective hydrochromic upconversion in Cs_3_GdCl_6_‐based materials as a mechanistically defined optical response rather than a simple wavelength‐dependent variation, and provides a design principle for excitation‐selective optical moisture readout.

## 2 Results

Cs_3_GdCl_6_ features a monoclinic structure characterized by a zero‐dimensional framework in which [GdCl_6_]^3−^ octahedra are isolated by Cs^+^ cations (Figure 1a). Within this lattice, two crystallographically distinct octahedral sites (Gd1 and Gd2) serve as ideal accommodation sites for lanthanide ion substitution, as shown in Figure 1b,c. The phase purity of the synthesized Cs_3_GdCl_6_ was verified by Rietveld refinement of the x‐ray diffraction (XRD) patterns. As shown in Figure 1d, the refinement results demonstrate excellent agreement between the observed and calculated profiles (χ^2^ = 1.826, R_wp_ = 7.63%), confirming a pure monoclinic structure free of secondary impurities. The detailed lattice parameters are listed in Table S1. Subsequently, Yb^3+^, Er^3+^, and Tb^3+^ were systematically introduced into the Cs_3_GdCl_6_ lattice, and their optical properties were investigated. The Cs_3_GdCl_6_ host was successfully doped with Yb^3+^, Er^3+^, or Tb^3+^. Consistent with Vegard's law [27], the unit cell volume contracted linearly as the dopant concentration increased (Figure 1e). This monotonic decrease in the lattice volume supports the substitution of Gd^3+^ ions with smaller lanthanide ions. The chemical compositions and electronic states of the materials were verified by x‐ray photoelectron spectroscopy (XPS). Each spectrum (Figure 1f) exhibits characteristic binding energies corresponding to the respective orbitals, indicating that the substituted ions are present in the trivalent oxidation state within the halide coordination environment. To optimize the luminescence efficiency following ion substitution in the Cs_3_GdCl_6_ system, a comparative analysis of the luminescence intensity for various compositions was performed. Cs_3_Gd_1‐x_Er_x_Cl_6_ exhibited the strongest luminescence intensity at an Er^3+^ concentration of 20 mol% (Figure S1a). Additionally, for the Cs_3_Gd_1‐x‐y_Yb_x_Er_y_Cl_6_ material with the introduced Yb^3+^ sensitizer, the strongest luminescence intensity was confirmed at an Er^3+^ concentration of 2 mol% under a fixed concentration of 20 mol% Yb^3+^ (Figures S1b and S2). Furthermore, in the Tb^3+^‐substituted composition, the maximum luminescence efficiency (excitation at 280 nm and emission at 548 nm) was observed at 25 mol% Tb^3+^ (Figures S1c and S3). Among these compositions, Cs_3_Gd_0.8_Er_0.2_Cl_6_ was selected for further investigation because it exhibited distinct emission behavior depending on the excitation wavelength.

**FIGURE 1:**
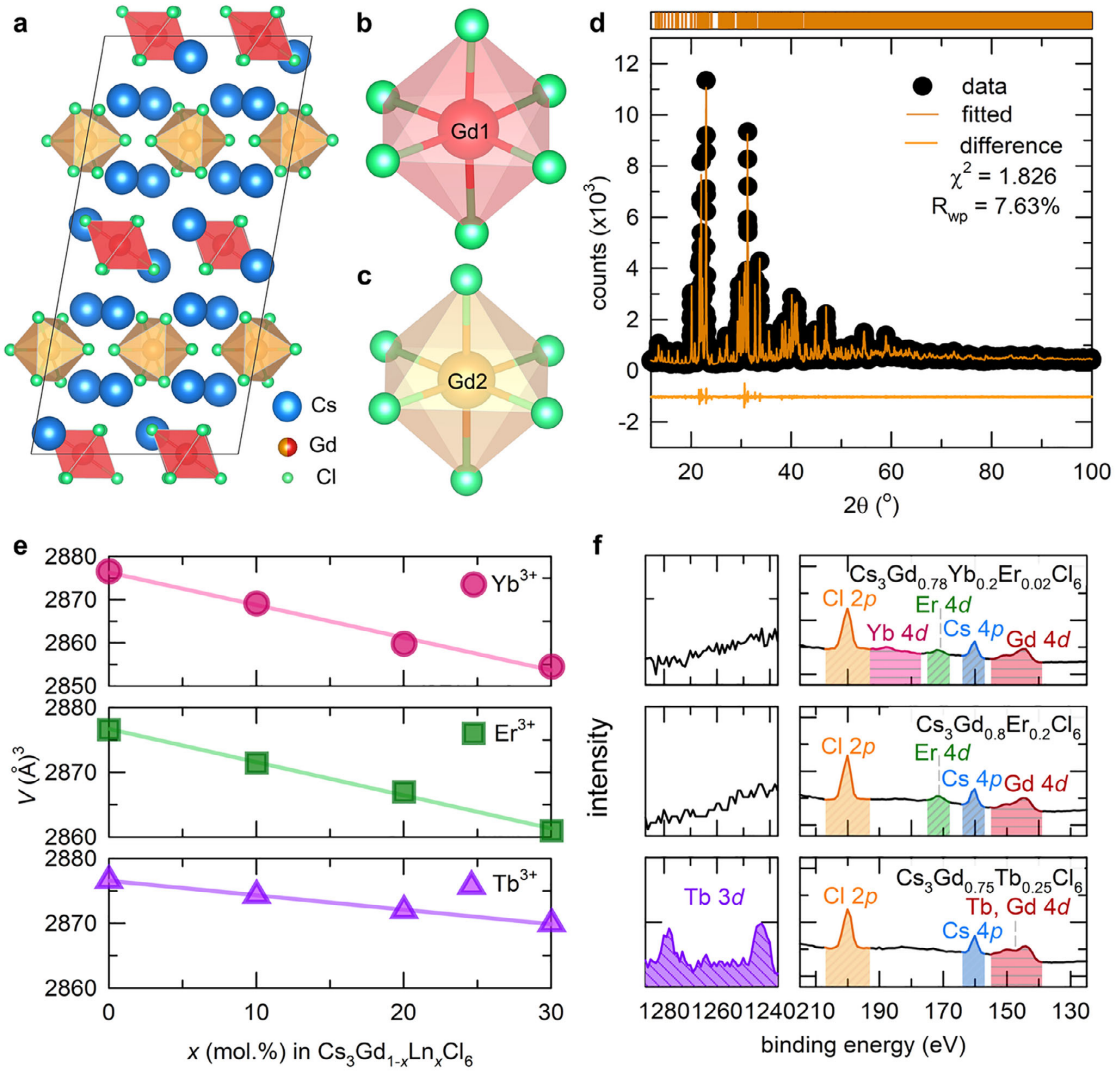
Structural analysis of Ln^3+^‐doped Cs_3_GdCl_6_. (a) Cs_3_GdCl_6_ crystal structure. (b) Gd1 and (c) Gd2 sites in GdCl_6_ octahedra. (d) Rietveld refined XRD pattern of Cs_3_GdCl_6_. (e) Cell parameters of Cs_3_Gd_1‐_ *_x_* Ln*_x_* Cl_6_ (Ln^3+^ = Yb^3+^, Er^3+^, Tb^3+^) were obtained after full‐pattern Rietveld refinement. (f) XPS survey spectra of Cs_3_Gd_1‐_ *_x_* Ln*_x_* Cl_6_.

As shown in Figure 2a,b, under 1550 nm laser excitation, green emission (525/550 nm) corresponding to the ^2^H_11/2_ → ^4^I_15/2_ and ^4^S_3/2_ → ^4^I_15/2_ transitions was dominant. In contrast, under 980 nm excitation, red emission (670 nm) originating from the ^4^F_9/2_ → ^4^I_15/2_ transition dominated. The specific excitation mechanisms of the upconversion photoluminescence are elucidated in the energy‐level diagrams of Figure 2c,d. Under 1550 nm excitation (Figure 2c), Er^3+^ ions undergo a three‐photon process involving the sequential absorption of three photons via ground‐state absorption (GSA) and excited‐state absorption (ESA).

**FIGURE 2:**
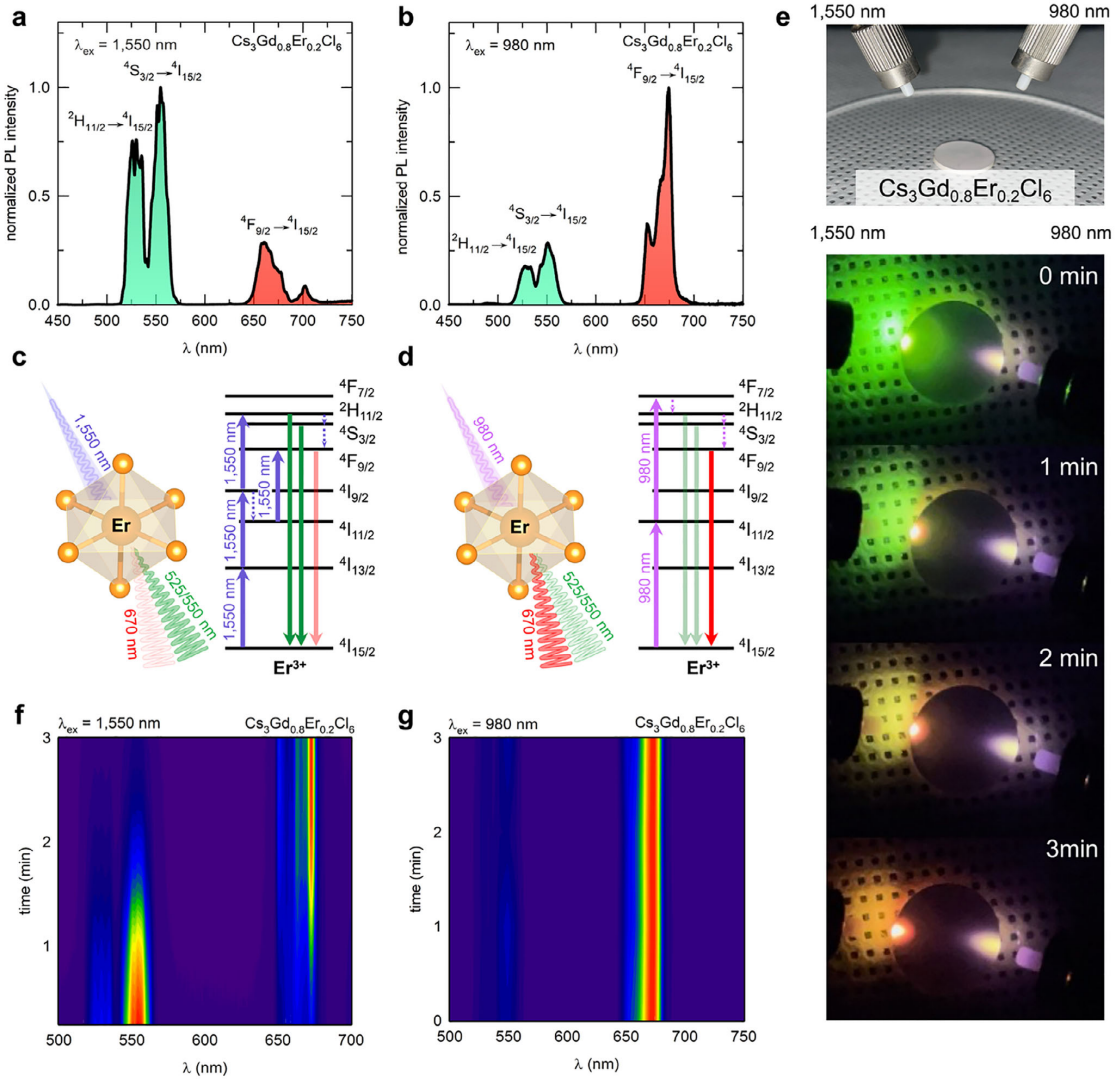
Excitation‐wavelength‐dependent upconversion and hydrochromic photoluminescence in Cs_3_Gd_0.8_Er_0.2_Cl_6_ (980 vs 1550 nm). PL spectra of Cs_3_Gd_0.8_Er_0.2_Cl_6_ under (a) 1550 nm and (b) 980 nm excitation. Upconversion photoluminescence scheme under (c) 1550 nm excitation and (d) 980 nm excitation. (e) Hydrochromic upconversion photoluminescence photographs of Cs_3_Gd_0.8_Er_0.2_Cl_6_: Exposure time in atmospheric moisture under 1550 and 980 nm excitation. Time‐dependent upconversion photoluminescence spectra under (f) 1550 nm and (g) 980 nm excitation of Cs_3_Gd_0.8_Er_0.2_Cl_6_.

Through this process, electrons are promoted to the upper ^2^H_11/2_ and ^4^S_3/2_ levels, and the subsequent radiative decay from these levels induces green and red emissions. On the other hand, under 980 nm excitation (Figure 2d), a two‐photon process acts as the primary pathway, where electrons absorb two photons to reach the ^4^F_7/2_ level. Electrons excited to this high energy level reach lower emissive levels via nonradiative relaxation, subsequently generating emissions at green and red wavelengths through electronic transitions.

To evaluate the dynamic optical response to moisture, the time‐dependent upconversion photoluminescence changes of Cs_3_Gd_0.8_Er_0.2_Cl_6_ were monitored at a relative humidity (RH) of 22%. Figure 2e presents photographs of the luminescence of the sample over time under 980 and 1550 nm laser excitation. Under 1550 nm excitation, the sample initially exhibited bright green luminescence but distinctly shifted to red within 3 min of exposure. In contrast, under 980 nm excitation, the sample displayed yellow luminescence, and no observable color change occurred during the same exposure period. Figure 2f shows the changes in the emission spectra under 1550 nm excitation. As the exposure time increased, the intensity of the green emission bands (centered at 525 and 550 nm) decreased rapidly, whereas the red emission component remained relatively dominant, resulting in an overall color transition from green to red. On the other hand, under 980 nm excitation (Figure 2g), the emission spectra remained virtually unchanged, maintaining constant luminescence characteristics throughout moisture exposure. These observations demonstrate that the occurrence of hydrochromic switching in this material depends on the excitation condition. As summarized in Figure S3, the PL intensity ratio I_550 nm_/I_670 nm_ decreases under both 980 and 1550 nm excitation for Cs_3_Gd_0.78_Yb_0.2_Er_0.02_Cl_6_, whereas for Cs_3_Gd_0.8_Er_0.2_Cl_6_ it decreases only under 1550 nm excitation and remains nearly unchanged under 980 nm excitation.

To elucidate the origin of the hydrochromic behavior under different excitation conditions, we performed a comparative analysis of the optical properties and upconversion mechanisms of Cs_3_Gd_0.78_Yb_0.2_Er_0.02_Cl_6_ and Cs_3_Gd_0.8_Er_0.2_Cl_6_ under 980 nm excitation. We previously reported that Cs_3_Gd_0.78_Yb_0.2_Er_0.02_Cl_6_ exhibits a distinct color transition from green to red upon moisture exposure under 980 nm irradiation [28]. In contrast, as observed in Figure 2, Cs_3_Gd_0.8_Er_0.2_Cl_6_ shows no color change under identical conditions. This discrepancy is attributed to the differences in the light absorption capabilities and the resulting upconversion pathways of the two materials.

Figure 3a,d compare the absorption spectra of the two samples. Cs_3_Gd_0.78_Yb_0.2_Er_0.02_Cl_6_ displays strong absorption bands at 980 nm, corresponding to the ^2^F_7/2_ → ^2^F_5/2_ transition of Yb^3+^ ions, and at 1550 nm, corresponding to the ^4^I_15/2_ → ^4^I_13/2_ transition of Er^3+^ (Figure 3a). This strong absorption enables efficient energy harvesting. On the other hand, Cs_3_Gd_0.8_Er_0.2_Cl_6_ exhibits weak absorption at 980 nm owing to the low oscillator strength of the ^4^I_15/2_ → ^4^I_11/2_ transition of Er^3+^, while showing a stronger absorption band at 1550 nm (^4^I_15/2_ → ^4^I_13/2_) (Figure 3d). This difference in absorption coefficients at 980 nm excitation plays a decisive role in determining moisture sensitivity. The kinetics of the upconversion process were further investigated using time‐resolved photoluminescence decay curves. For Cs_3_Gd_0.78_Yb_0.2_Er_0.02_Cl_6_, a clear population buildup phase was observed in the green emission (550 nm) decay curve prior to moisture exposure (t = 0 min) (Figure 3b). This indicates that a rise time is required for the energy absorbed by the Yb^3+^ ions acting as sensitizers to be transferred to the Er^3+^ ions and for the population in the emissive state to accumulate, which is an inherent kinetic characteristic of the energy transfer upconversion (ETU) mechanism. However, as the moisture exposure time increased from 0 to 8 min, this rise‐time characteristic gradually disappeared, and the overall lifetime decreased significantly. This implies that the high‐energy OH^−^ oscillations of absorbed water molecules effectively quench the ^4^S_3/2_ and ^2^H_11/2_ levels of Er^3+^, thereby hindering ETU efficiency. Meanwhile, the decay of the red emission (670 nm) showed relatively little change compared to the green emission, resulting in the dominance of the red emission (Figure 3c). In stark contrast, Cs_3_Gd_0.8_Er_0.2_Cl_6_ exhibited distinctly different decay kinetics under 980 nm excitation. As shown in Figure 3e,f, no population buildup was observed in either the green or red emission decay curves. The absence of such a rise time suggests that without the Yb^3+^ sensitizer, the upconversion process in Cs_3_Gd_0.8_Er_0.2_Cl_6_ proceeds primarily via GSA, followed by ESA, rather than through ETU. Importantly, the luminescence decay curves of Cs_3_Gd_0.8_Er_0.2_Cl_6_ remained unchanged throughout the 8‐min moisture exposure. These decay characteristics corroborate the results shown in Figure 2g, confirming that the ESA‐mediated yellow emission pathway of the Cs_3_Gd_0.8_Er_0.2_Cl_6_ system exhibited no color change due to moisture under 980 nm excitation. Based on these observations, we propose the mechanistic models illustrated in Figure 3g,h. In Cs_3_Gd_0.78_Yb_0.2_Er_0.02_Cl_6_, the high absorption coefficient of Yb^3+^ facilitates efficient ETU, populating the green‐emitting levels of Er^3+^. However, these populated levels are highly susceptible to nonradiative relaxation caused by the high‐energy OH^−^ oscillations (oscillation quenching, OQ) of adjacent water molecules, leading to a hydrochromic shift from green to red emission. In contrast, as depicted in Figure 3h, the Cs_3_Gd_0.8_Er_0.2_Cl_6_ system under 980 nm excitation exhibits a limited population density owing to its low absorption coefficient, and the emission mechanism relies primarily on ESA. This pathway inherently favors stable red emission and is less affected by multiphonon relaxation induced by surface‐adsorbed water, thereby maintaining spectroscopic stability. Consistent with this surface‐mediated picture, Raman spectra recorded during moisture exposure under three excitation wavelengths show that the two vibrational bands of the [GdCl_6_]^3−^ octahedra, at 124 and 264 cm^−1^, retain their position and linewidth, while the Er^3+^ emission in the same spectra changes markedly (Figure S15 and Table S3). The absence of any shift or broadening indicates that the hydrochromic response is not accompanied by lattice disorder or defect formation. To confirm the hypothesis that hydrochromic behavior is fundamentally governed by the absorption coefficient, we further compared the responses under 1550 nm excitation (Figure S5). Unlike the 980 nm excitation, where only Cs_3_Gd_0.78_Yb_0.2_Er_0.02_Cl_6_ exhibited strong absorption, 1550 nm excitation directly initiated the ^4^I_15/2_ → ^4^I_13/2_ transition of Er^3+^ ions; thus, both samples possess high absorption coefficients regardless of the presence of Yb^3+^. Consistent with our expectations, both samples exhibited a rapid hydrochromic color transition from green to red upon moisture exposure under 1550 nm excitation. This contrasts with the results obtained under 980 nm excitation (Figure 2g) and supports the view that sufficiently strong absorption, enabling the population of moisture‐sensitive high‐energy levels, is required for hydrochromic switching. The same conclusion holds at the level of excited‐state dynamics. Whereas the decay curves of Cs_3_Gd_0.8_Er_0.2_Cl_6_ remain invariant under 980 nm excitation (Figure 3e,f), time‐resolved measurements of the same material under 1550 nm excitation, monitored at 525, 550, and 670 nm and referenced to a vacuum‐sealed state, show that the lifetimes of the green‐emitting levels decrease more rapidly than that of the red‐emitting level throughout the exposure (Figure S13). The excitation‐selective character of the response is therefore reproduced in the decay kinetics and not only in the steady‐state color. While vibrational quenching by adsorbed water is the dominant contribution to this behavior, a secondary role for moisture‐induced changes in Er^3+–^Er^3+^ cross‐relaxation cannot be excluded.

**FIGURE 3:**
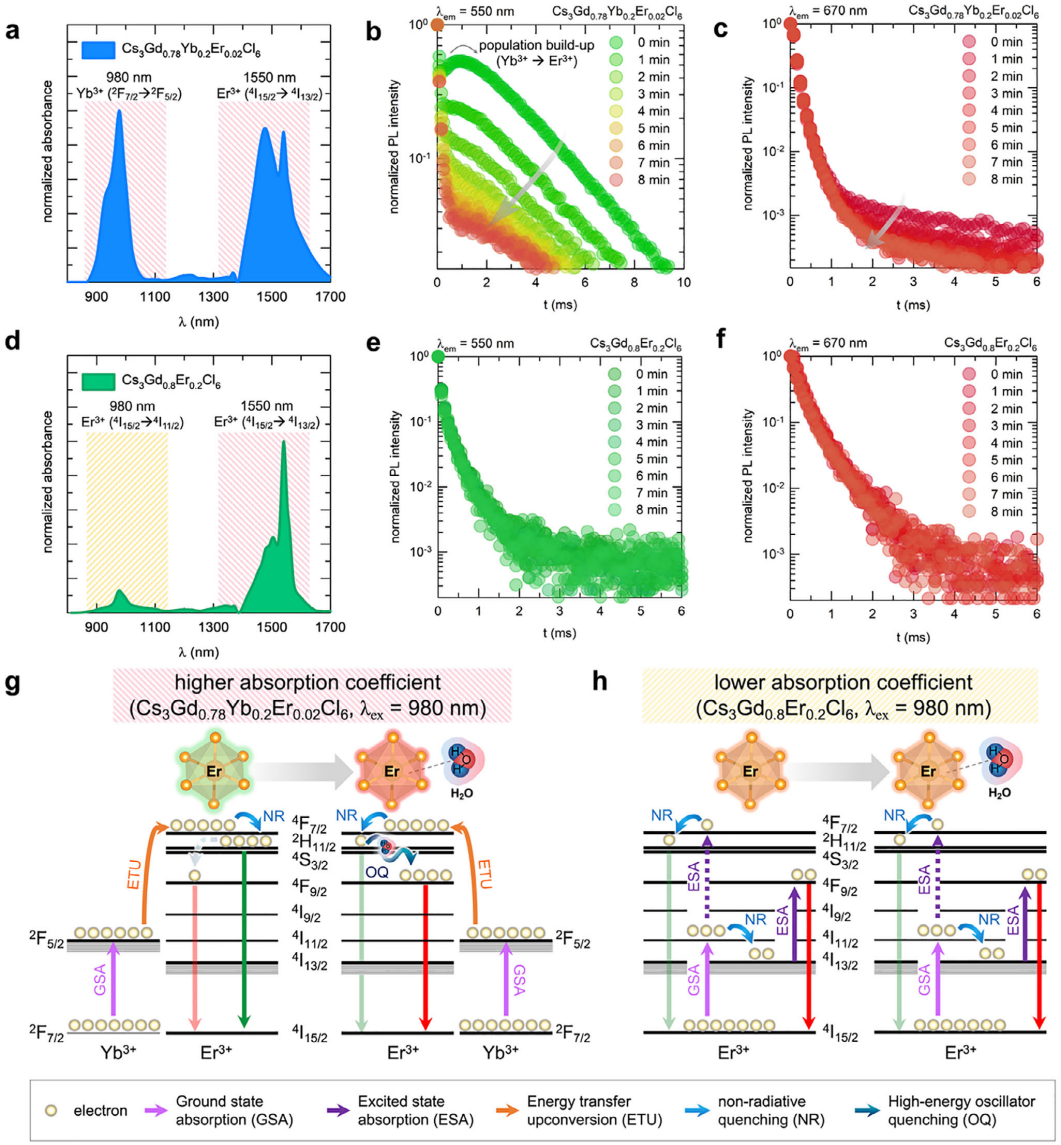
Spectroscopic and kinetic evidence for hydrochromic upconversion pathways: Yb/Er co‐doped vs Er‐only systems. (a) Normalized absorbance spectra of Cs_3_Gd_0.78_Yb_0.2_Er_0.02_Cl_6_. Time‐resolved photoluminescence decay curves of Cs_3_Gd_0.78_Yb_0.2_Er_0.02_Cl_6_ monitored at (b) 550 nm and (c) 670 nm. (d) Normalized absorbance spectra of Cs_3_Gd_0.8_Er_0.2_Cl_6_. Time‐resolved photoluminescence decay curves of Cs_3_Gd_0.8_Er_0.2_Cl_6_ monitored at (e) 550 nm and (f) 670 nm. Schematic illustration of hydrochromic upconversion mechanism under 980 nm excitation for (g) Cs_3_Gd_0.78_Yb_0.2_Er_0.02_Cl_6_ and (h) Cs_3_Gd_0.8_Er_0.2_Cl_6_.

To further validate the upconversion mechanisms, we investigated the pump power dependence of the luminescence intensity (I ∝ P^n^). Under 980 nm excitation, the intensity slope of approximately 2 confirmed a two‐photon process, whereas intensity slopes between 2.5 and 3.0 under 1550 nm excitation indicated a three‐photon process (Figure S6). A direct comparison reveals that the luminescence intensity of Cs_3_Gd_0.78_Yb_0.2_Er_0.02_Cl_6_ at 980 nm is nearly two orders of magnitude higher than that of the Er^3+^‐singly doped sample (Figure S2). This drastic difference reflects the weak absorption at 980 nm of Er^3+^, which restricts the excited‐state population. Consequently, this limited population prevents effective moisture‐induced quenching, resulting in a stable yellow emission. Quantifying this difference from the diffuse‐reflectance spectra, the within‐spectrum absorbance ratio A(1550 nm)/A(980 nm) is 3.11 for Cs_3_Gd_0.8_Er_0.2_Cl_6_ but inverts to 0.56 for Cs_3_Gd_0.78_Yb_0.2_Er_0.02_Cl_6_, and the hydrochromic response is absent only in the condition with the weakest absorption at the excitation wavelength (Figure S3 and Table S4). We note that the apparent rate of the transition additionally reflects the order of the upconversion pathway, since a higher‐order process passes through more intermediate states susceptible to OH^−^ quenching; the absorption at the excitation wavelength governs whether the response occurs rather than how fast the ratio evolves.

To examine additional optical functions of the Ln^3+^‐substituted compositions, we evaluated their temperature‐dependent upconversion luminescence properties in the range of 20°C–200°C. As clearly illustrated in the contour maps in Figure S7, most compositions and excitation conditions (980/1550 nm excitation for Cs_3_Gd_0.8_Er_0.2_Cl_6_ and 980 nm excitation for Cs_3_Gd_0.78_Yb_0.2_Er_0.02_Cl_6_) exhibited typical thermal quenching behavior, where the overall luminescence intensity rapidly decreased with increasing temperature because of the activation of nonradiative relaxation induced by lattice vibrations. However, under 1550 nm excitation, Cs_3_Gd_0.78_Yb_0.2_Er_0.02_Cl_6_ displayed distinctly different spectral evolution (Figure S8a). Specifically, the emission intensities at 525 nm (^2^H_11/2_ → ^4^I_15/2_) and 550 nm (^4^S_3/2_ → ^4^I_15/2_) exhibited different thermal responses (Figure S8b,c). While the 550 nm emission intensity decreased markedly as the temperature increased from 293 to 473 K, the 525 nm emission showed a much smaller variation over the same temperature range. As a result, the relative contribution of the 525 nm emission increased with temperature, leading to an increase in the fluorescence intensity ratio. This phenomenon is attributed to thermal population redistribution following the Boltzmann distribution between the thermally coupled levels of ^2^H_11/2_ and ^4^S_3/2_. Analysis of the fluorescence intensity ratio (FIR, I_525_/I_550_) of these two transitions yielded a well‐fitted curve (Figure S8d), confirming its validity as a ratiometric optical thermometer. The calculated maximum relative sensitivity (S_r_) was 0.75% K^−1^, demonstrating that this material also shows temperature‐dependent optical response.

Additionally, the Tb^3+^‐substituted composition Cs_3_Gd_0.75_Tb_0.25_Cl_6_ showed efficient luminescence properties under both x‐ray and ultraviolet (UV) excitations. Radioluminescence (RL) measurements revealed the characteristic 548 nm emission peak of Tb^3+^ ions, which is identical to the photoluminescence characteristics obtained under UV excitation (Figure S4). As shown in Figure S9a, this green emission spectrum matches well with the spectral sensitivity region of commercial CMOS image sensors. This high spectral matching implies that the sensor efficiency can be maximized by converting the photon signals generated by the scintillator into electronic signals, making it highly advantageous for applications in indirect x‐ray imaging systems. The RL intensity also showed a linearly increasing trend with varying x‐ray dose rates (Figure S9b). This linearity suggests that quantitative detection is possible over a wide range of x‐ray exposure, from low to high doses.

Using the Cs_3_GdCl_6_‐based compositions examined in this study, we present two application approaches: qualitative visualization and quantitative analysis. Figure 4 illustrates the implementation of a pattern plate that qualitatively demonstrates optical pattern readout through immediate and intuitive visual responses to various external stimuli. We fabricated the pattern plate by arranging Cs_3_Gd_0.75_Tb_0.25_Cl_6_, Cs_3_Gd_0.78_Yb_0.2_Er_0.02_Cl_6_, and Cs_3_Gd_0.8_Er_0.2_Cl_6_ in a specific configuration (Figure 4a). The fabricated pattern plate conceals the encoded pattern under natural daylight because the hidden patterns are completely indistinguishable to the naked eye in natural daylight (Figure 4b). However, upon the application of external stimuli, this pattern plate exhibits different optical responses depending on the stimulus, as shown in Figure 4c, thereby qualitatively visualizing hidden information. First, under 254 nm UV irradiation, Cs_3_Gd_0.75_Tb_0.25_Cl_6_ positioned at the periphery was excited, revealing a vivid green circular pattern (Figure 4d). Using the same principle, when the plate was placed on top of a CMOS sensor array and exposed to x‐rays, a circular pattern was imaged at an identical location (Figure 4e). This demonstrates stimulus‐dependent optical readout under UV and x‐ray excitation.

**FIGURE 4:**
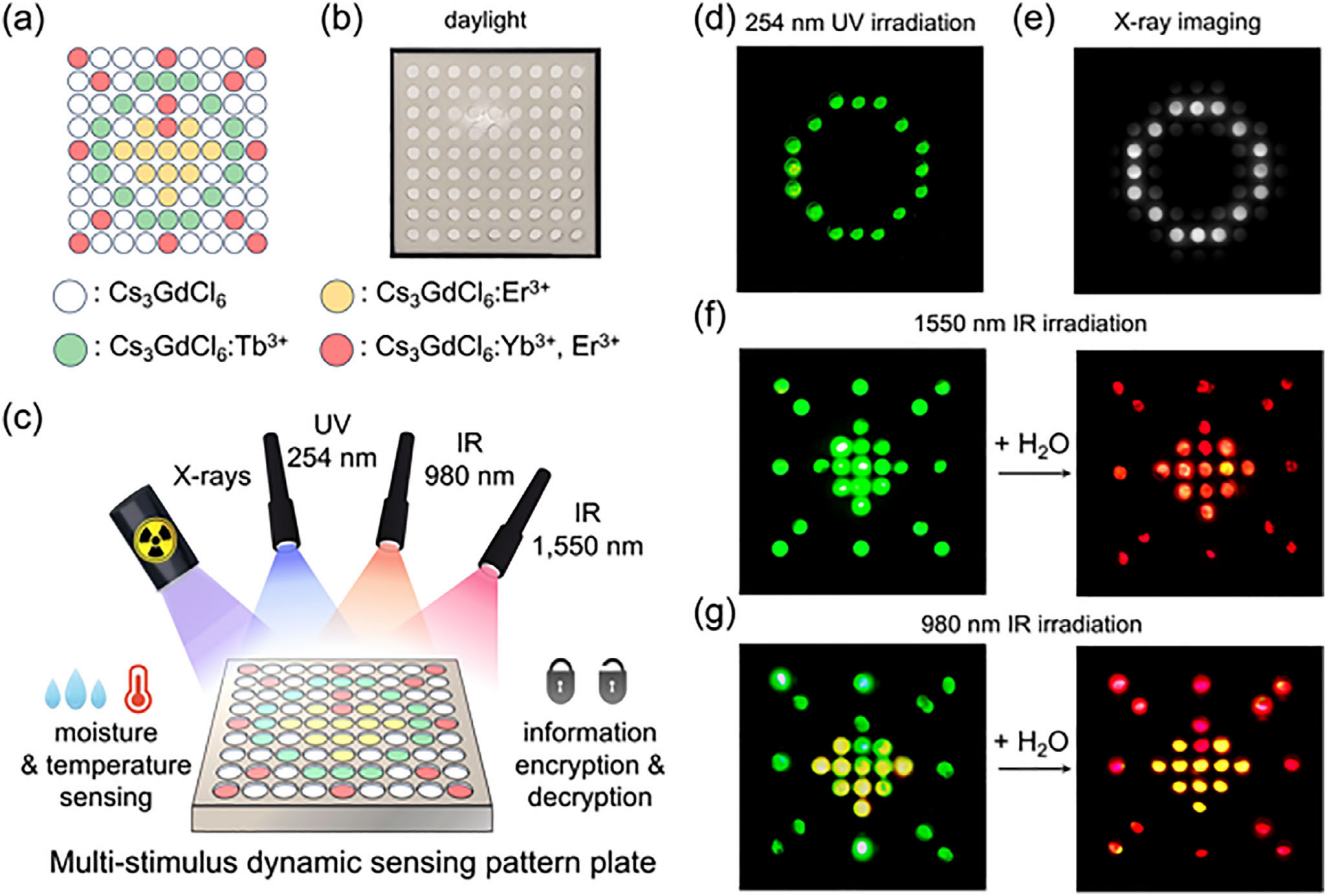
Qualitative stimulus‐dependent optical pattern readout using Cs_3_GdCl_6_‐based compositions. (a) Schematic design of the pattern. (b) Photograph under daylight. (c) Schematic illustration of optical readout under different external stimuli. (d) Green circular pattern under 254 nm excitation. (e) x‐ray image of the circular pattern. (f) Pattern images under 1550 nm excitation before and after moisture exposure. (g) Pattern images under 980 nm excitation before and after moisture exposure.

In particular, long‐exposure imaging using 1550 and 980 nm infrared (IR) lasers clearly exhibits the excitation‐dependent optical readout and moisture response of the pattern plate. Under 1550 nm excitation in a dry environment, both Cs_3_Gd_0.78_Yb_0.2_Er_0.02_Cl_6_ and Cs_3_Gd_0.8_Er_0.2_Cl_6_ emitted green light, revealing a central green comet‐like pattern (Figure 4f). Upon exposure to atmospheric moisture in this state, the green emission is quenched, whereas the red emission dominates owing to the high moisture sensitivity of both materials under 1550 nm excitation, resulting in the entire pattern transitioning to red. This provides a qualitative visual indication of moisture exposure through color change.

On the other hand, distinctly different behavior was observed under 980 nm laser excitation. Under 980 nm excitation, the luminescence of Cs_3_Gd_0.8_Er_0.2_Cl_6_ becomes prominent, forming a yellow, heart‐shaped pattern (Figure 4g). Cs_3_Gd_0.8_Er_0.2_Cl_6_ maintains spectroscopic stability against moisture under 980 nm irradiation; thus, the heart pattern retains its yellow hue without significant color alteration, even after moisture exposure. Because this pattern is insensitive to moisture while the 1550 nm pattern is not, reading the same plate under both excitations provides an internal reference that separates the moisture response from any general change in the emission. These results show that different optical information can be selectively visualized within a single pattern plate depending on excitation condition and moisture exposure.

We further explored the potential of Cs_3_Gd_0.8_Er_0.2_Cl_6_ for quantitative moisture analysis in sealed systems that require nondestructive optical readout. Its rapid and pronounced color evolution under 1550 nm excitation enables high‐sensitivity tracking of accumulated moisture exposure through time‐dependent optical signals. On this basis, we designed a power‐free sticker‐type humidity dosimeter by combining the 1550 nm optical‐key feature with quantitative image‐based analysis.

To overcome the limitations of qualitative visual observation and to precisely elucidate the reaction kinetics, we constructed an automated analysis framework that extracts frames from video data, automatically detects the region of interest (ROI), and converts the emission colors into a CIE L*a*b* coordinate system (Figure 5a). To ensure the accuracy of quantitative analysis, it is essential to clearly define the starting and ending points of the reaction. The a* value of the material gradually increased with continued moisture exposure and reached an equilibrium state without further changes once the interior of the crystal lattice became saturated with moisture. Based on this saturation behavior, we established the boundary conditions. Specifically, by defining the a* value in the initial moisture‐free state (OH^−^ 0%) as the minimum and the a* value at equilibrium in the moisture‐saturated state (OH^−^ 100%) as the maximum (Figure 5a, right), we normalized the entire reaction progress to a saturation level ranging between 0% and 100%. In this framework, the a* value serves as a quantitative indicator of the green‐to‐red hydrochromic transition. The a* value of the moisture‐free state (−33.21) and that of the equilibrium moisture‐saturated state (20.37) define two fixed boundary states, and the measured a* value at any given time indicates where the current state lies between these two limits.

**FIGURE 5:**
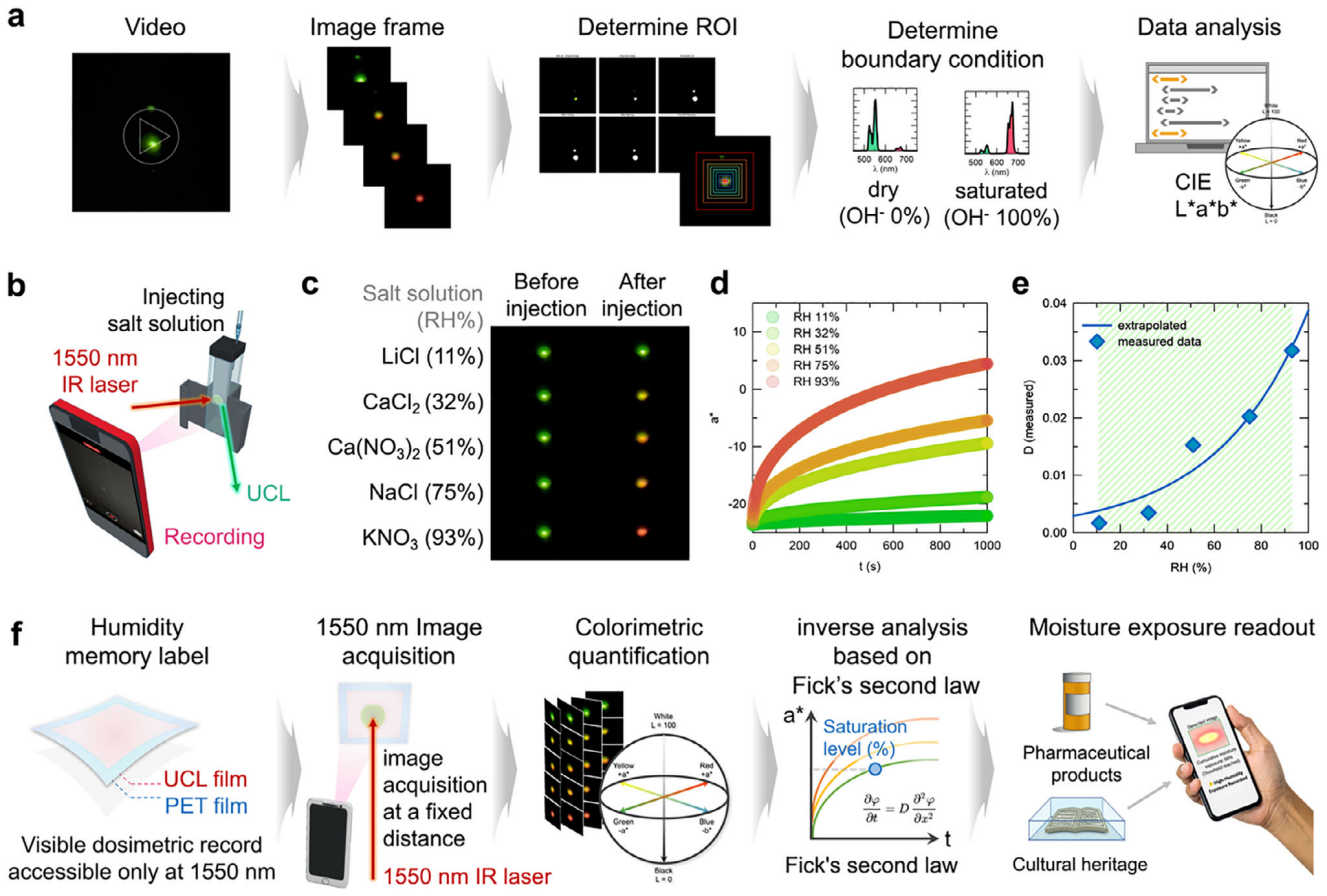
Humidity dosimetry quantification and diffusion‐based readout. (a) Video data analysis framework for determining saturation level. (b) Experimental setup and (c) dosimeter images at various RH values. (d) Time‐resolved a* value evolution. (e) Diffusion coefficient versus RH. (f) Smartphone‐based dosimetric readout system based on Fick's second law.

Experiments were conducted under various RH environments precisely controlled from 11% to 93% using saturated salt solutions (Figure 5b,c), allowing for real‐time tracking of color changes due to moisture infiltration. The fitted time‐resolved response curves in Figure 5d show the changes in a* values according to humidity conditions, with the corresponding time‐dependent spectra given in Figure S10. Under all humidity conditions, the a* values tended to increase over time and converged to the set saturation boundary value, and the rate of change increased exponentially in higher‐humidity environments. Recognizing that this behavior was a physical phenomenon of moisture molecules diffusing into the crystal lattice, we fitted the data using the long‐time approximation solution of Fick's second law [29, 30]. The experimental data showed very high agreement (R^2^ > 0.97) with the theoretical model under high‐humidity conditions, and the derived diffusion coefficients (D) across the entire humidity range exhibited an exponentially increasing trend with a high correlation coefficient of R^2^ > 0.96 with respect to the relative humidity (Figure 5e). Fitting statistics for all measured humidity conditions are summarized in Table S2, and the accuracy of the inverse humidity readout is retained across the full range (Figure S14). This indicates that the material exhibits predictable moisture‐response behavior over a continuous range, rather than acting only as a simple discoloration indicator.

Based on the established model, we propose a smartphone‐based dosimeter reading system equipped with an inverse analysis based on Fick's second law (Figure 5f). Unlike conventional binary indicators such as CoCl_2_ [31, 32], this system quantifies the color coordinates of images captured under a 1,550 nm laser and performs an inverse calculation by substituting them into a master curve. Thus, it is possible to derive the saturation level, which represents the current accumulated moisture exposure state, regardless of fluctuating exposure rates. This is because the measured a* value does not simply reflect the instantaneous external humidity at the moment of readout, but rather the extent of moisture that has already diffused into the material relative to the two fixed boundary states. Accordingly, the saturation level provides a readout of the accumulated moisture uptake stored in the dosimeter, even when the exposure rate varies over time. By providing not only simple warnings but also quantitative values (e.g., saturation level: 68%) through a smartphone interface, it offers an effective solution for the nondestructive diagnosis of internal moisture integrity using only an attached sticker for sealed packaging systems, where external interference must be minimized. These characteristics demonstrate the potential of Cs_3_GdCl_6_‐based materials as optical moisture indicators for environments requiring controlled humidity management, such as precision chemicals, pharmaceutical storage, and cultural heritage management. The device is designed to record cumulative moisture exposure rather than instantaneous humidity, and its readout is stable under prolonged 1550 nm irradiation in a dry environment (Figure S12); regeneration of the assembled sticker is accordingly not part of its intended operation, although the luminescent material itself recovers after thermal treatment (Figure S11).

## 3 Conclusions

In this study, we demonstrated excitation‐selective hydrochromic upconversion in Cs_3_Gd_0.8_Er_0.2_Cl_6_. Under the same moisture‐exposure condition, hydrochromic switching was absent under 980 nm excitation but occurred distinctly under 1550 nm excitation. Spectroscopic and kinetic analyses showed that this contrast originates from excitation‐pathway‐dependent population of moisture‐sensitive excited states and is closely linked to the absorption coefficient at the excitation wavelength. A sufficiently high absorption coefficient enables the population of moisture‐sensitive high‐energy levels, which are subsequently quenched from green to red emission by the high‐energy OH‐ oscillations of adjacent water molecules. These findings establish that the occurrence of hydrochromic switching is determined by whether the excitation condition can sufficiently populate moisture‐sensitive excited states, thereby establishing a mechanistic basis for excitation‐selective hydrochromic response in upconversion materials. On the basis of this mechanism, we further implemented qualitative stimulus‐dependent optical readout and a power‐free sticker‐type moisture dosimeter capable of quantitative analysis of accumulated moisture exposure based on Fick's second law. Taken together, these findings establish an excitation‐selective switching criterion for hydrochromic upconversion in zero‐dimensional metal halides and provide a design principle for qualitative optical readout and quantitative moisture dosimetry.

## 4 Materials and Methods

### 4.1 Materials

All reagents were used without purification. Cesium chloride (CsCl, 99.9%), and gadolinium chloride hexahydrate (GdCl_3_∙6H_2_O, 99.9%) and other lanthanide precursors (LnCl_3_∙6H_2_O, 99.9%; Ln = Tb, Er, Yb) were purchased from Sigma–Aldrich. All chemicals were used without any further purification.

### 4.2 Synthesis of Cs_3_Gd_1‐_ *_x_* Ln*_x_* Cl_6_

All chemicals were used without any further purification. Stoichiometric amounts of starting materials were ground in agate mortar, placed in alumina crucibles, and fired at 450°C for 24 h in a tubular furnace using Ar gas.

### 4.3 Structural and Morphology Characterizations

The structures of the as‐synthesized samples were characterized by XRD (Miniflex 600) using a diffracted beam monochromator set for Cu‐Kα radiation (λ = 1.54056 Å). The 2θ scan range was 10°–100° with a step size of 0.01°. Structural information was derived from Rietveld refinement using the GSAS software suite [33]. A three‐dimensional visualization system for electronic and structural analysis (VESTA) was used to draw the crystal structures [34]. The phase purity of the as‐synthesized samples was estimated via Rietveld refinement of the XRD results with the consideration of full refinement of the crystallographic and instrumental parameters in the GSAS program suite. XPS (K‐Alpha) was used to analyze the chemical composition of the samples.

### 4.4 Optical Characterizations

Steady‐state PL spectra of the samples were recorded using a Hitachi F‐7000 fluorescence spectrophotometer. Upconversion luminescence properties of the samples were characterized using 980 nm laser diode (IRM980TA‐1000FC, Shanghai Laser & Optics Century) and 1550 nm laser diode (EB21387, CNI Laser). The variation in the PL intensity during heating was measured by connecting a Hitachi F‐7000 fluorescence spectrometer to an integrated heater, temperature controller, and thermal sensor. Raman spectra were acquired under 455, 532, and 785 nm excitation, with the sample held at a fixed position throughout the exposure sequence (DXR3xi, Thermo Fisher). Time‐resolved photoluminescence decay curves under 1550 nm excitation were recorded at 525, 550, and 670 nm; the sample was measured first in a vacuum‐sealed state and then at the same position after opening to ambient laboratory air at 50% RH. Thermal treatment for recovery experiments was performed at 150°C for 30 min inside a glovebox.

### 4.5 Controlled Humidity Environments

Humidity‐dependent kinetic experiments were conducted inside a custom‐designed sealed chamber. Precise control over the relative humidity (RH) was achieved using various saturated salt solutions at room temperature. Specifically, Saturated Lithium Chloride (LiCl), Calcium Chloride (CaCl_2_), Calcium Nitrate (Ca(NO_3_)_2_), Sodium Chloride (NaCl), and Potassium Nitrate (KNO_3_) solutions were prepared to establish RH levels of 11%, 32%, 51%, 75%, and 93%, respectively.

### 4.6 Automated Video Processing and Region of Interest Detection

To minimize observer bias and ensure high‐throughput analysis of the moisture reaction, we developed a custom automated analysis pipeline using Python (v3.11). Video recordings of the sensor response under controlled relative humidity (RH) conditions were processed using the OpenCV library, where files were decomposed into individual frames at a sampling rate of 1 Hz to generate a time‐series image dataset. To automatically identify the active crystal area, we implemented an adaptive detection algorithm that first converts the initial frame to grayscale and generates a binary mask using a dynamic threshold. This threshold was determined as the median of three statistical parameters derived from pixel intensity distributions: the mean intensity plus one standard deviation, 95% of the 85th percentile intensity, and twice the mean intensity. Following morphological operations to remove noise, a fixed window of 50 × 50 pixels was established around the centroid of the detected sensor contour to ensure consistent spatial sampling across all timeframes.

### 4.7 Colorimetric Analysis and Kinetic Modeling

For quantitative kinetic analysis, the RGB values within the defined ROI were averaged and converted to the CIE L*a*b* color space using the skimage.color module, specifically tracking the a* coordinate which indicates the green‐red color transition. The raw a* values were normalized to a saturation percentage (S(t)) ranging from 0% to 100% based on pre‐determined boundary conditions, where the values ‐33.21 and 20.37 were defined as the initial anhydrous state and the fully saturated equilibrium state, respectively. The resulting time‐resolved saturation data were analyzed using the long‐time approximation of Fick's second law.

$$\frac{\partial C}{\partial t}=D\frac{\partial^{2}C}{\partial x^{2}}$$

For the long‐time approximation, the experimental data were fitted to the following exponential solution using the Levenberg‐Marquardt algorithm [35] via the SciPy library:

$$S\left(t\right)=S_{\infty }-\left(S_{\infty }-S_{0}\right)\;\exp\left(-Dt\right)$$

This exponential model provided a superior fit (R^2^ > 0.97) for high humidity conditions compared to square‐root models, allowing for the precise extraction of diffusion coefficients (D) to characterize the moisture infiltration kinetics.

## Author Contributions

**Joo Hyeong Han**: conceptualization, writing – original draft, investigation. **Jeong Min Seo**: investigation, data curation. **Sung Woo Jang**: data curation, investigation. **Yong Min Park**: investigation. **San Ha Choi**: investigation. **Ji Hyeon Cha**: validation. **Won Bin Im**: conceptualization, writing – review and editing, supervision.

## Conflicts of Interest

The authors declare no conflicts of interest.

## Supporting information




**Supporting File**: advs78059‐sup‐0001‐SuppMat.docx.

## Data Availability

The data that support the findings of this study are available from the corresponding author upon reasonable request.
