# Supplementary material for: Excitation‐Selective Hydrochromic Upconversion in Zero‐Dimensional Metal Halides
Source: Adv Sci (Weinh). 2026 Sep 28:e78059. Online ahead of print. doi: 10.1002/advs.78059 (PMC13618766; doi:10.1002/advs.78059)
Supplement: Supplementary file 1 [file ADVS-9999-e78059-s001.docx]

**Supporting Information**

**Excitation-selective hydrochromic upconversion in zero-dimensional metal halides**

**Joo Hyeong Han, Jeong Min Seo, Sung Woo Jang, Yong Min Park****, San Ha Choi, Ji Hyeon Cha, and Won Bin Im***

*Division of Materials Science and Engineering, Hanyang University, 222 Wangsimni-ro, Seongdong-gu, Seoul, 04763, Republic of Korea*

*****To whom correspondence should be addressed.

E-mail: [imwonbin@hanyang.ac.kr](mailto:imwonbin@hanyang.ac.kr)

**Table S1.** Rietveld refinement parameters of combined X-ray diffraction profiles of Cs_3_GdCl_6_. The numbers in parentheses are the estimated standard deviations of the last signiﬁcant ﬁgure.

| parameters | value |
| --- | --- |
| symmetry | monoclinic |
| space group | *C*2/*c* |
| *a* (Å) | 27.025(8) |
| *b* (Å) | 8.172(2) |
| *c* (Å) | 13.235(9) |
| *α* (°) | 90.000 |
| *β* (°) | 100.094(2) |
| *γ* (°) | 90.000 |
| *V* (Å^3^) | 2877.568(2) |
| *R_wp_* (%) | 7.63 |
| *χ*^2^ | 1.826 |

**Table S2.** Diffusion coefficients and fitting statistics obtained from the humidity-response kinetics of Cs_3_Gd_0.8_Er_0.2_Cl_6_ at each relative humidity, evaluated over a common 1250 s window. The dynamic range refers to the total change in the CIE a* value over that window.

| RH (%) | a* dynamic range | D | R^2^ |
| --- | --- | --- | --- |
| 11 | 1.90 | 0.0017 | 0.857 |
| 32 | 5.04 | 0.0035 | 0.747 |
| 51 | 15.37 | 0.0153 | 0.905 |
| 75 | 19.19 | 0.0203 | 0.970 |
| 93 | 29.25 | 0.0318 | 0.976 |

**Table S3.** Peak position and full width at half maximum of the two vibrational Raman bands of Cs_3_Gd_0.8_Er_0.2_Cl_6_ under 455 nm excitation, obtained by Gaussian fitting after subtraction of a local linear background. Values are given for the as-prepared sample and after thermal treatment at 150 °C for 30 min in a glovebox. Uncertainties are the standard errors of the fit.

| Sample | Exposure (min) | 124 cm^−1^ position | 124 cm^−1^ FWHM | 264 cm^−1^ position | 264 cm^−1^ FWHM |
| --- | --- | --- | --- | --- | --- |
| as-prepared | 0 | 124.4 ± 0.4 | 23.5 ± 1.0 | 264.5 ± 0.1 | 14.2 ± 0.2 |
| as-prepared | 2 | 124.3 ± 0.4 | 23.1 ± 1.0 | 264.3 ± 0.1 | 14.2 ± 0.2 |
| as-prepared | 4 | 124.2 ± 0.4 | 23.3 ± 0.9 | 264.3 ± 0.1 | 14.0 ± 0.2 |
| as-prepared | 6 | 124.8 ± 0.4 | 23.3 ± 1.0 | 264.4 ± 0.1 | 14.3 ± 0.3 |
| after 150 °C | 0 | 125.0 ± 0.6 | 23.2 ± 1.4 | 264.5 ± 0.1 | 13.3 ± 0.1 |
| after 150 °C | 2 | 124.2 ± 0.4 | 23.2 ± 1.0 | 264.2 ± 0.1 | 14.0 ± 0.2 |
| after 150 °C | 4 | 124.2 ± 0.4 | 23.6 ± 1.0 | 264.3 ± 0.1 | 14.2 ± 0.1 |
| after 150 °C | 6 | 124.5 ± 0.5 | 24.0 ± 1.1 | 264.3 ± 0.1 | 14.1 ± 0.2 |

**Table S4.** Absorbance at the excitation wavelength and hydrochromic response for the four measured conditions. A(1550)/A(980) is the absorbance ratio within each spectrum: a value above 1 means that 1550 nm is the more strongly absorbed of the two wavelengths, and a value below 1 that 980 nm is. The I_550_/I_670_ ratio, its change and the time to 90% of the total change (t_90_) are from the time-dependent measurements under identical exposure conditions (22% RH, Figure S3).

| Composition | Excitation | A(1550)/A(980) | I_550_/I_670_  (initial → final) | Change | t_90_ |
| --- | --- | --- | --- | --- | --- |
| Cs_3_Gd_0.8_Er_0.2_Cl_6_ | 980 nm | 3.11 | 0.18 → 0.22 | none | – |
| Cs_3_Gd_0.8_Er_0.2_Cl_6_ | 1550 nm | 3.11 | 3.95 → 0.09 | −98% | 3 min |
| Cs_3_Gd_0.78_Yb_0.2_Er_0.02_Cl_6_ | 980 nm | 0.56 | 3.95 → 0.29 | −93% | 19 min |
| Cs_3_Gd_0.78_Yb_0.2_Er_0.02_Cl_6_ | 1550 nm | 0.56 | 3.94 → 0.09 | −98% | 8 min |

**Figure S1.** Normalized PL intensity of Cs_3_Gd_1-_*_x_*Ln*_x_*Cl_6_ as a function of (a) Er^3+^, (b) Yb^3+^-Er^3+^, and (c) Tb^3+^ concentration.

**Figure S2.** PL spectra under 980 nm and 1550 nm excitation of Cs_3_Gd_0.78_Yb_0.2_Er_0.02_Cl_6_.

**Figure S3.** Time dependent change in the PL intensity ratio I_550 nm_/I_670 nm_ of Cs_3_Gd_0.78_Yb_0.2_Er_0.02_Cl_6_ and Cs_3_Gd_0.8_Er_0.2_Cl_6_ under 980 nm and 1550 nm excitation during moisture exposure. Measurements were performed under ambient conditions at 22% RH, with the sample held in place throughout the exposure.

**Figure S4.** PL and PLE spectra of Cs_3_Gd_0.75_Tb_0.25_Cl_6_.

**Figure S5.** Schematic illustration of hydrochromic upconversion mechanism under 1550 nm excitation for Yb/Er co-doped and Er-doped systems.

**Figure S6.** Laser power-dependent PL spectra under 980 nm and 1550 nm excitation of (a-d) Cs_3_Gd_0.8_Er_0.2_Cl_6_ and Cs_3_Gd_0.78_Yb_0.2_Er_0.02_Cl_6_. Pump power dependence of green and red upconversion intensity: (e-h) Cs_3_Gd_0.8_Er_0.2_Cl_6_ and Cs_3_Gd_0.78_Yb_0.2_Er_0.02_Cl_6_under 980 nm and 1550 nm excitation.

**Figure S7.** Temperature-dependent upconversion luminescence contour maps of Cs_3_Gd_0.8_Er_0.2_Cl_6_ and Cs_3_Gd_0.78_Yb_0.2_Er_0.02_Cl_6_ under 980 nm and 1550 nm excitations in the temperature range of 20–200℃.

**Figure S8.** Optical thermometry performance of Cs_3_Gd_0.78_Yb_0.2_Er_0.02_Cl_6_ under 1550 nm excitation. (a) Comparison of absorbance slopes, (b) Temperature-dependent PL spectra showing thermally coupled level behavior, (c) Temperature-dependent peak intensity variations of the 525 nm and 550 nm emissions. (d) fluorescence intensity ratio (FIR) fitting and relative sensitivity (S_r_) curve.

**Figure S9.** X-ray detection characteristics of Cs_3_Gd_0.75_Tb_0.25_Cl_6_. (a) Spectral matching between radioluminescence (RL) emission and the spectral sensitivity of a commercial CMOS sensor. (b) Linear relationship between RL intensity and X-ray dose rate.

**Figure S10.** Time-dependent hydrochromic upconversion spectra and corresponding luminescence images of Cs_3_Gd_0.8_Er_0.2_Cl_6_ under 1550 nm excitation at different relative humidity conditions controlled by saturated salt solutions.

**Figure S11.** Reversibility of the hydrochromic response of Cs_3_Gd_0.8_Er_0.2_Cl_6_ under ambient air and near-saturated humidity exposure. (a) PL spectra under ambient air exposure and after subsequent thermal treatment. (b) PL spectra under near-saturated humidity exposure and after subsequent thermal treatment. (c) Corresponding XRD patterns for Figure S11a. (d) Corresponding XRD patterns for Figure S11b.

**Figure S12.** Time-dependent CIE a* values and representative luminescence images of Cs_3_Gd_0.8_Er_0.2_Cl_6_ under continuous 1550 nm irradiation in a dry, very low-humidity environment.

**Figure S13.** Time-resolved photoluminescence of Cs_3_Gd_0.8_Er_0.2_Cl_6_ under 1550 nm excitation, monitored at 525, 550 and 670 nm. (a) Mono-exponential lifetimes against real time; the shaded region is the vacuum-sealed state before exposure and the dotted line marks opening to ambient air at 50% RH. (b) Relative change with respect to the vacuum reference. (c) Expanded view of the first 8 min, with the interval of the green-to-red color transition shaded. The sample was measured at a fixed position throughout. Error bars are the standard error of the fit.

**Figure S14.** Humidity-response kinetics of Cs_3_Gd_0.8_Er_0.2_Cl_6_ evaluated over a common 1250 s window. (a) Dynamic range of the CIE a* response, (b) coefficient of determination of the diffusion fit and (c) absolute error of the relative humidity obtained by inverse prediction; the dashed line marks the mean error of 1.1%. The lower R^2^ at low humidity reflects the small dynamic range of the response rather than a failure of the model.

**Figure S15.** Raman spectra of Cs_3_Gd_0.8_Er_0.2_Cl_6_ recorded during moisture exposure at 50% RH, at a fixed sample position. (a) Spectra under 455 nm excitation after 0, 2, 4 and 6 min, each normalized to its own maximum. (b) Position and full width at half maximum of the 264 cm^−1^ band, measured on the as-prepared sample and after thermal treatment at 150 °C for 30 min in a glovebox. (c) Spectra under 455 and 785 nm excitation on a common Raman-shift axis; the bands at 124 and 264 cm^−1^ coincide and are therefore vibrational in origin. (d) The same spectra, with those under 532 nm excitation, against absolute wavelength; the Er^3+^ emission manifolds are shaded and the dotted lines mark the two vibrational bands.
